# Thermal, Morphological and Mechanical Properties of a BioPE Matrix Composite: Case of Shell, Pulp, and Argan Cake as Biofillers

**DOI:** 10.3390/ma16062241

**Published:** 2023-03-10

**Authors:** Jihane Zeghlouli, Nicola Schiavone, Haroutioun Askanian, Amine Guendouz, Cherkaoui El Modafar, Philippe Michaud, Cédric Delattre

**Affiliations:** 1 Centre d’Agrobiotechnologie et Bioingénierie, Unité de Recherche Labellisée CNRST (Centre AgroBiotech, URL-CNRST 05), Faculté des Sciences et Techniques Marrakech, Université Cadi Ayyad, Marrakech 40000, Morocco; 2 Clermont Auvergne INP, CNRS, Institut Pascal, Université Clermont Auvergne, F-63000 Clermont-Ferrand, France; 3 Institut de Chimie de Clermont Ferrand (ICCF), UMR 6296 Université Clermont Auvergne, CNRS, SIGMA Clermont, F-63000 Clermont-Ferrand, France; 4 Institut Universitaire de France (IUF), 1 Rue Descartes, 75005 Paris, France

**Keywords:** argan byproducts, biocomposite, extrusion, bio-polyethylene

## Abstract

Extrusion and hot compressing molding processes were used to create bio-polyethylene (BioPE) composites reinforced with argan byproducts (shell, pulp, and argan cake) as bio-fillers. The thermal stability of the composites wass analyzed by differential scanning calorimetry (DSC) and thermogravimetric analysis (TGA). Dynamical mechanical analysis and rheological testing were used to investigate their mechanical properties. The morphological results showed a good adhesion between the argan and BioPE matrix. More efficient mechanical properties have been distinguished in the case of argan byproduct-based composite. A higher Young’s modulus was noted for all the biocomposites compared to pure BioPE. Thermal analysis revealed that the addition of bio-filler to polymer reduced decomposition temperatures. This study provides an ecological alternative for upgrading the valorization of abundant and underutilized Moroccan biomass. Furthermore, the possibility of using argan byproducts in composite manufacturing will help open up new markets for what is currently considered waste.

## 1. Introduction

Petro-plastics are among the main materials used in the preparation of products with a short lifespan, particularly in the packaging industry [1,2]. This generates a large amount of plastic waste, which has been causing global environmental problems for decades [3,4,5,6,7]. A massive amount of plastic waste has been released into the environment all over the world, contributing to the current problem of white pollution [8]. As a result, the negative effects of plastics on society have prompted the scientific community, governments, and businesses to seek solutions to these problems.

Recycling, incineration, and landfilling are the current methods for managing plastic waste [1]. However, these steps are often limited by some issues related to the high cost of the process, the low cost of recycled plastics, the low cost of raw materials limiting plastic recycling, and the production of greenhouse gases and other irritant gases [3,4]. Also, the efficient of these methods remains very limited and only validated under certain conditions, because the scale of plastic production far outnumbers the reduction achieved by these treatment methods, and more work needs to be done on awareness of responsible waste handling methods [5].

In this respect, nature-based, renewable, and biodegradable composite represent a viable alternative to petro-plastics and present a topic of great interest [6]. Additionally, to address the issues caused by industrial waste, alternative products are being developed in collaboration with the recycling sector and renewable resources, particularly biosourced composites, which are critical in creating an environmentally friendly, economical, and sustainable raw material source [7,8,9,10,11,12,13].

Biocomposites can be created by combining biowastes, additives, polymers, and fillers with varying properties and putting them through a variety of processes. These composites can be given the desired properties based on their intended use, or new products can be improved [14]. Furthermore, numerous biopolymers such as, PLA, PHAs, protein, and starch-based plastics are being researched as potential alternatives to petro-plastics in combating the ongoing plastics problem [15,16].

Biocomposites made from biomass-based renewable resources are expected to become an indispensable alternative in the future [17]. The advantages of plant-derived composites include their biodegradability in nature and waste recycling by the environment. However, polymer composites reinforced with natural fillers from renewable natural resources, known as “Green biocomposites”, present a topic of great interest. They provide numerous benefits, such as renewability, non-toxicity, biodegradability, low density, improved thermal and acoustic insulating properties, low energy consumption during processing, low cost, etc. [18,19,20,21,22].

Many studies have been conducted to evaluate bio-fillers in order to develop composites with improved performance [23,24]. The incorporation of lignocellulosic fiber as reinforcement phases into post-consumer polymers (as matrices) improves their properties and thus broadens their applications. As a result, this method minimizes the environmental impact of waste materials [25,26,27]. Different lignocellulosic fiber can be acquired from forestry and agricultural resources (e.g., jute, sisal, hemp, kenaf, wood, cotton, flax, bagasse, etc.) [28,29], from agricultural wastes or byproducts (e.g., rice husks, coir, wheat bran, okra, pineapple, and artichoke) [30,31,32,33], and also from particles such as almond shells, almond kernels, and pistachio and peanut shells [34,35,36,37]. However, agricultural wastes are more interesting as fillers due to their ease of processing and low cost to produce as alternative, cheap, sustainable, and environmentally safe materials for several sectors. For these reasons, there is always an interest in finding new natural fibers as reinforcement of composites from agricultural waste.

**Figure 1:**
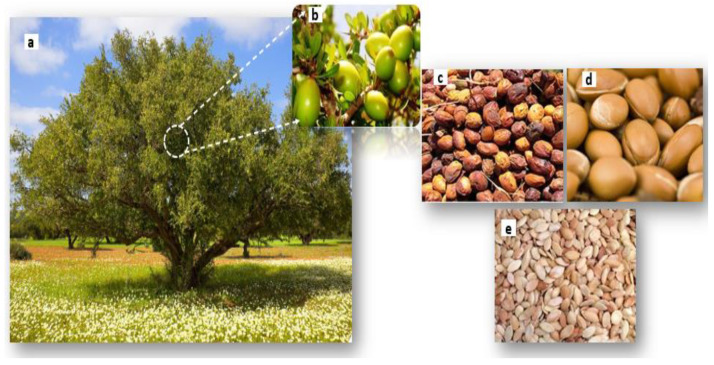
The argan tree (Argania spinosa (L) Skeels) and its different parts: (**a**) tree, (**b**) immature fruits, (**c**) pulp, (**d**) shell, (**e**) kernels.

The argan byproducts could have a promising future as a new bio-filler. The argan tree (*Argania spinosa* (L.) Skeels) exclusively grows endemically in Morocco, which represents its second forest, with about 871,210 hectares, 20 million trees, and a production of 500 kg of fruit per ha per year [38]. For centuries, it played a crucial role in the ecology and the socio-economy of the country, taking into account that 3.5 million local lives depend on the argan ecosystem [39]. This illustrious species is designated as a UNESCO biosphere reserve and is universally known for its nutritional, therapeutic, and cosmetic oil [39]. However, the production process of argan oil generated a large quantity of byproducts representing about 97% of the argan fruit weight (43% of pulp, 52.6% of shells, and 2% of argan cake generated after the extraction of oil from argan kernels) (Figure 1) which should be better exploited. So far, the main use of argan shell is for heating, while the pulp and argan cake are used as cattle feed [40].

Recently, some studies on the use of argan shell as a bio filler in different matrices were reported [41,42,43,44]. However, up to now, and to the best of our knowledge, there is no study that has evaluated the valorization of all argan byproducts (shell, pulp, and cakes) as bio filler, which could have the privilege of boosting the valorization of the local byproducts of the argan tree, with the goal of establishing an argan biorefinery and to contribute to the economic development of the argan region.

In this work, our main goal was to produce and analyze a new bio polyethylene (BioPE) based composite with argan byproducts as filler. We chose this type of polyethylene because it has the same chemical structure as petro-based polyethylene and is widely accessible on an industrial scale [45].

We investigated the impact of adding each argan byproduct (30 wt.%) to BioPE grades on the composite films. The thermal, rheological, morphological, and mechanical properties were assessed.

## 2. Materials and Methods

### 2.1. Raw Materials

Argan byproducts (shell, pulp, and two types of argan cakes) utilized in this study were supplied by Nectarome (https://www.nectarome.com/ accessed on 10 December 2019), located 35 km from Marrakech (Morocco). It represents one of the leading companies in Morocco for the production of oil and cosmetic products based on argan. We mentioned that the two types of argan cakes studied, roasted and unroasted, represent the first and second extractions from the production of edible and cosmetic oils, respectively. The company had already dried and ground the samples of argan byproducts into a fine powder. Following that, the biomass was stored until the day of use.

The content of main chemical constituents of the three argan byproducts is shown in Table 1. Argan byproduct analysis was performed using the methods highlighted in our previous study [46]. Braskem (So Paulo, Brazil) supplied the matrix, which was bio-based bimodal high-density polyethylene (HDPE) SGE 7252 with a density of 0.952 g/cm^3^ and a melt flow index (190 °C/2.16 kg) of 2 g/10 min.

### 2.2. Laser Granulometry Measurement

The samples grain size, size–volume distribution %, and the specific surface area, were measured through Laser granulometry technique based on light diffraction with a Mastersizer Laser Diffraction granulometry analyzer from Malvern Analytical (Mastersizer 3000, Malvern Instruments Co. Ltd., Malvern, UK). The samples were individually measured in a liquid bulk container, and the mixing process was conducted in water with sonification to eliminate the probability of agglomeration of the particles. Mastersizer software was used to analyze the results.

### 2.3. Composite Film and Filament Preparation for 3D Printing

A Thermo Fisher Scientific Pharma twin screw extruder was used to prepare composite films with argan byproduct powder (30 wt.%) (Waltham, MA, USA). Before starting the extrusion step, the bio-PE pellets and argan byproducts were dried in a vacuum oven for 24 h at 60 °C. Each single byproduct shell, pulp, or argan cake was mixed with 70% weight mass of BioPE. The extrusion conditions were 150 °C, 100 rpm, and 0.6 Kg/h for temperature, screw rate, and mass flow, respectively.

**Figure 2:**
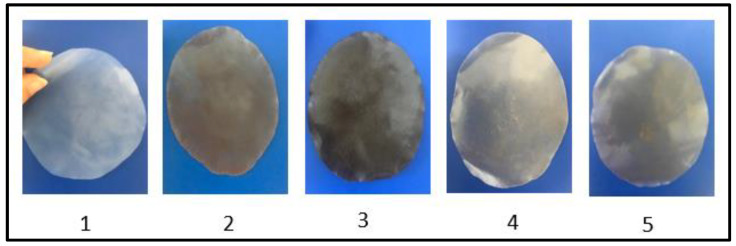
Preparation of composite. 1: BioPE 100%; 2: BioPE-30% shell; 3: BioPE-30% pulp; 4: BioPE-30% unroasted argan cake; 5: BioPE-30% roasted argan cake.

The wire was then pressed for 2 min (150 °C, 3 MPa) using hot press (Weber Pressen Hy-draulic Press, WEBER-HYDRAULI K, Güglingen, Germany). Compression molding of BioPE pellets (control) and BioPE-argan byproducts composite between Teflon^®^-lined mild steel plates produced a thin film approximately 0.1 mm thick. These processes produced thin films measuring 5 cm × 4 cm. Those films (Figure 2) were used for thermal, morphological, and mechanical characterization.

### 2.4. Rheological Testing

Shear dynamic measurements were done with an ARES rheometer (Rheometric Scientific) made by TA Instruments (New Castle, DE, USA) using a frequency sweep method and parallel plate geometry with an 8 mm diameter. The temperature of the test was 150 °C, the gap was placed to 1 mm, 5% of strain was performed, and a sweep frequency of 0.1100 rad/s was set. The calculation of the loss viscosity (η′) and the storage viscosity (η″) was made from the frequencies and the zero-shear viscosity η0, as described in a previous study [47].

### 2.5. Thermal Characterization

A thermogravimetric analysis was performed on all samples to verify the impact of the load of argan byproducts on the thermal properties of the polymer and to determine which of the byproducts, shell, pulp, or argan cakes, is the most thermally stable.

#### 2.5.1. Thermogravimetric Analyses (TGA)

TGA was carried out on a PerkinElmer TGA 4000 (Waltham, MA, USA) with a heating rate of 10 °C/min. In an aluminum pan, 8 mg of the sample was heated from room temperature to 700 °C under a nitrogen atmosphere.

#### 2.5.2. Differential Scanning Calorimetry (DSC) Analysis

Under a nitrogen atmosphere, the crystallinity and thermal behavior of the analyzed samples were assessed using differential scanning calorimetry (METTLER TOLEDO DSC 3+, Columbus, OH, USA). An 8–10 mg mass of material was tested for each sample using the heat–cool–heat cycle method described by Schiavone et al., 2021 [48].

The crystallization degree X_c_ was determined using the following equation:

X_u_ = (ΔH_m_ − ΔH_c_)/(ΔH_m0_ − (w/w) p) × 100

with ΔH_m_, ΔH_m0_, and ΔH_c_ as the melting enthalpy; the melting enthalpy of pure, fully crystalline polymers; and the crystallization enthalpy, respectively; (w/w) p as the polymer mass fraction in the matrix; and ΔH_m0_ = 287.7 J/g for PE.

### 2.6. Mechanical Characterization

**Figure 3:**
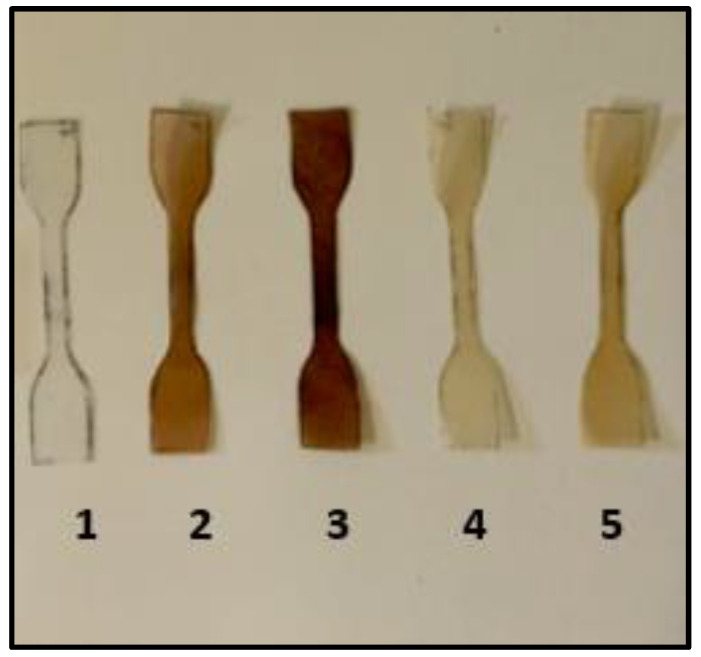
Preparation of composite specimens. 1: BioPE 100%; 2: BioPE-30% shell; 3: BioPE-30% pulp; 4: BioPE-30% unroasted argan cake; 5: BioPE-30% roasted argan cake.

The tensile properties were determined utilizing a Universal Testing Machine (Hounsfield, Model H50 Ks 0404, UK) with a cross-head speed of 50 mm/min. The specimens (Figure 3) measured 75 ± 0.2 mm in length, 5 ± 0.1 mm in width, and 0.2 ± 0.05 mm in thickness. The tests were carried out at a temperature of 25 °C. Data reported were the means of five replicates.

## 3. Results and Discussion

### 3.1. Granulometry Distribution

**Figure 4:**
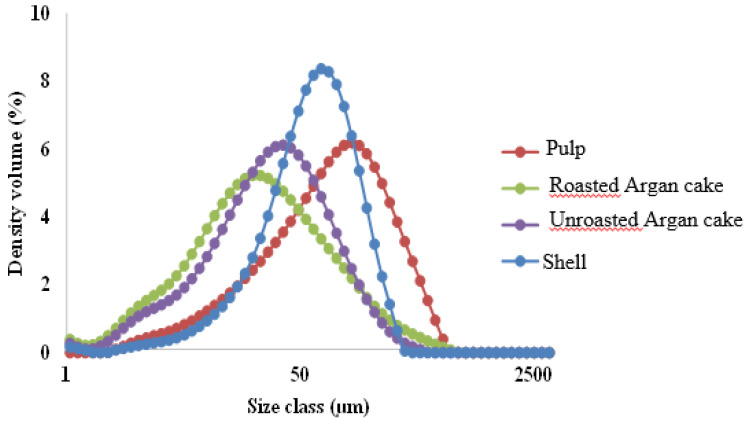
Particle size distribution of argan byproduct granules.

The granulometry of argan byproduct granules was determined using a laser granulometry technique (Figure 4, Table 2). The sizes at 10 (D10), 50 (D50), 90y (D90), and D [4;3] (volume mean diameter) of cumulative volume were calculated. The low granulometry was obtained in the case of roasted cakes; the obtained values were 5.6 μm, 29.2 μm, 95.0 μm, and 43.3 μm for D10, D50, D90 and D [4;3], respectively. However, the pulp predefined the sample with the largest particle size with 19.3 μm, 101.0 μm, 301.0 μm, and 135.0 μm for D10, D50, D90, and D [4;3], respectively. Additionally, the specific surface area was 559.1 cm^2^/g and 139.0 cm^2^/g for roasted argan cake and pulp, respectively. The distribution lacked dispersibility, and the size of the narrow distribution (span) for roasted argan cake and pulp, respectively, was 3.1 and 2.8, calculated by dividing the difference in diameter at 90% and 10% of the cumulative volume by the diameter at 50% of the cumulative volume [49].

### 3.2. Composite Thermal and Rheological Properties

#### 3.2.1. Analysis of Differential Scanning Calorimetry (DSC)

**Figure 5:**
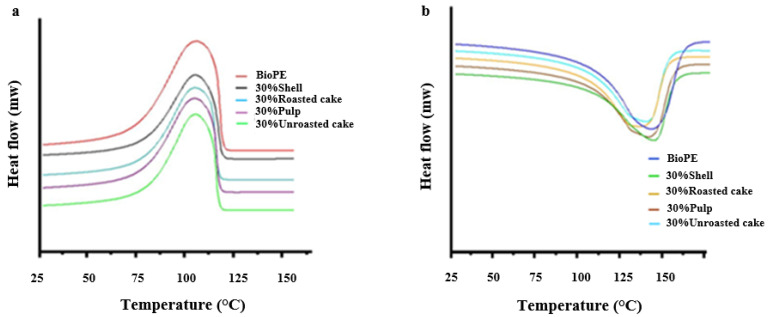
Differential scanning calorimetry: (**a**) second heating thermogram, (**b**) second cooling thermogram.

Differential scanning calorimetry was used to investigate the impact of argan byproduct on the thermal characteristics of BioPE composites. The second heating and cooling DSC thermograms of pure BioPE and BioPE/argan byproduct composites are shown in Figure 5a,b. The curves depicted do not show significant distinctions in terms of melting and cooling transitions; we obtained the same trend curve, approximatively. The thermal parameters, including melting enthalpy (ΔH_m_), melting temperature (T_m_), crystallization temperature (T_c_), crystallization enthalpy (ΔH_c_), and crystallinity degree (X_c_) are stated in Table 3. The adding of argan byproducts had no effect on the glass transition and melting temperatures of the biocomposites studied. However, we noticed a reduction in melting enthalpy (ΔH_m_) and crystallization enthalpy (ΔH_c_) values in the presence of argan by product composite compared to pure BioPE.

The melting temperature of neat Bio-HDPE is 133 °C, the melting enthalpy is 111 j/g, and the crystallinity XC is 39.6%. Schiavone et al. [48] reported comparable results. Melting enthalpy decreases slightly after the addition of argan particles to the Bio-PE, falling to 73 j/g, 56 j/g, 32 j/g, and 31 j/g for shell, pulp, roasted argan cake, and unroasted argan cake composite, respectively. We suggest that the reduction of melting enthalpy can be an advantage for ease of extrusion during 3D printing of the composite, which represents the last stage of valuation of a composite. Less thermal energy allows a rapid phase transition of the polymer filament compared to the residence of the material in the extruder [50,51]. On the other hand, crystallinity drops dramatically to 23.1%, 25.9%, 27.6%, and 30.5% for shell, pulp, roasted argan cake, and unroasted argan cake composite, respectively. This was related to the decrease in polymer chain mobility. In addition, while argan particles can stimulate heterogeneous nucleation during the polymer’s crystallization process, increased argan byproduct concentrations (over 10%) can make concessions to the nucleation due to particle–particle contact. As a result, there is a limitation in space for crystal formation and growth [52,53]. Essabir et al. [41] also noticed this phenomenon.

#### 3.2.2. Thermogravimetric Analysis (TGA)

**Figure 6:**
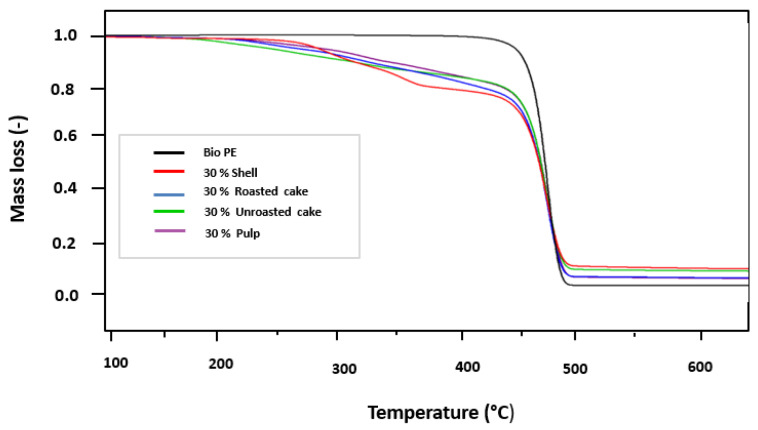
Thermogravimetric analysis thermogram.

Thermogravimetric analysis (TGA) has been investigated. Figure 6 depicts TGA curves for all composites in the presence of various argan byproducts. As can be seen, the maximum degradation rate temperature in the case of pure BioPE was 480 °C. For pure Bio-PE, Montanes et al. [54] discovered a comparable thermal profile.

However, the presence of fillers significantly influenced the decomposition kinetics. In other words, using argan byproducts as fillers appears to have reduced the initial degradation temperature. We discovered that the decomposition of argan byproduct-based composites begins around 220 °C. This is primarily due to the presence of argan byproducts in the polymer structure, which reduces thermal stability in some ways. Essabir et. al. [36] investigated a similar study on the properties of polypropylene and almond shell flour blends. Jorda-Reolid et al. [53] recently reported on the properties of plastic composites based on argan shell as a filler and high-density polyethylene in the presence of different compatibilizers. Contrary to the pure PE, the biocomposites based on argan byproducts curves show several stages of degradation. This can be explained by the degradation of the three major components of argan byproducts, namely, hemicellulose, cellulose, and lignin, as shown in Table 1 [36,55,56].

#### 3.2.3. Rheology

**Figure 7:**
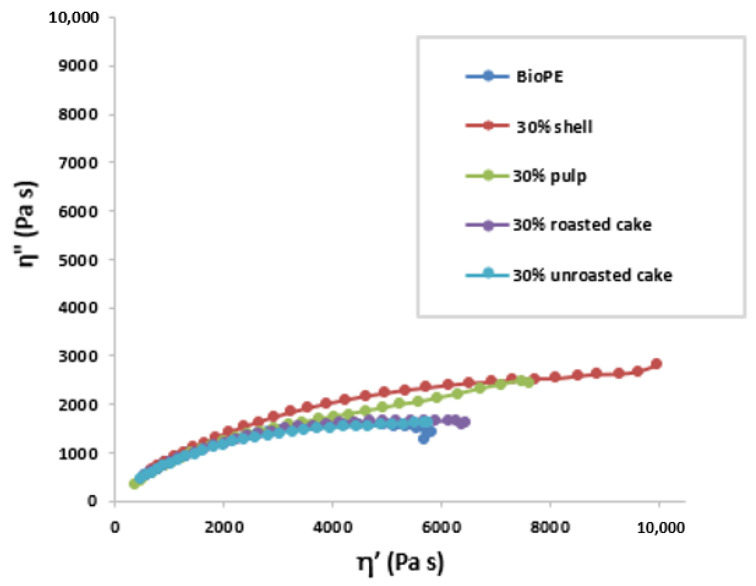
Cole–Cole plot for all the composites studied.

Melt viscoelasticity experiments in oscillatory shear mode with a rotational controlled stress rheometer were used to investigate the effect of argan byproduct as a filler content on the viscoelastic properties of the composites. Figure 7 depicts the Cole–Cole plots for all samples. The Cole–Cole distribution reveals a significant increase in zero-shear viscosity by adding argan byproduct as a filler, especially in the case of argan shell, as shown in the results. The increased effect of viscosity after the addition of the filler is normally due to the restricted mobility of chain segments, which alters the normal polymer flow [57,58]. In addition, the good distribution and good affinity of rigid bio-filler into the polymer increases its resistance to flow and subsequently results in an increase in viscosity [59,60]. Therefore, the presence of argan byproducts enhances viscoelastic properties and demonstrates good interfacial interaction between the argan byproduct fillers and the BioPE matrix. Moreover, the dynamic rheological properties of treated argan shell bio-filler reinforced high density polyethylene matrix were assessed in order to determine the dispersion state of the bio-filler within the polymer [61]. The results showed a raise in complex viscosity with the incorporation of bio-filler, and the composite containing the highest load of bio filler (25%) led to the highest complex values of viscosity. Moreover, the formulations of composites produced with pozzolan as a filler and bio-based polyethylene have been reported [48]. There was a proportional increase in viscosity with increasing pozzolan loading.

### 3.3. Mechanical Characteristics

Table 4 displays the mechanical properties of pure BioPE and BioPE/argan byproduct composites, including Young’s modulus (E), maximum tensile strength (max), ultimate strain (max), and elongation at break (b).

These findings are important for assessing the efficacy of various argan byproducts in terms of Bio-PE resistance improvement and compatibility with argan byproduct particles.

The films obtained by hot press showed an increase in stiffness by adding argan byproduct (30 wt.%) in the polymer matrix, characterized by an increase in Young’s modulus and a slight decrease in ultimate strength and ultimate strain.

Incorporation of shell, pulp, roasted argan cake, and unroasted argan cake (30 wt.%) into the BioPE matrix increased the Young modulus up to 498 MPa, 349 MPa, 324 MPa, and 330 MPa, respectively. This can be explained by the distribution of argan byproduct particles in the polymer matrix, which results in good filler–polymer adhesion and a more rigid material [62]. The higher Young’s modulus in the case of argan shell (498 MPa) could be explained by the complex composition of this material compared to the pulp and cakes shown by a high percentage of cellulose, hemicellulose, and lignin (Table 1).

However, there is a significant reduction in elongation at break, which can be attributed to the use of an excess of argan byproduct, which increases the stiffness of the material [63]. Furthermore, the presence of a filler causes phase heterogeneity and discontinuity in the polymer matrix. In general, this discontinuity reduces ductility [64,65].

Moreover, Essabir et al. [61] noted the same behavior using HDPE with argan particles, in that Young’s modulus increased with MAS concentration, but tensile strength began to decrease as argan shell particle load increased. In 2021, Schiavone et al. [53] also obtained improved stiffness from BioPE and pozzolane-based composites. The modulus of elasticity of the composites was three times that of pure BioPE, and the ability to print BioPE with filler mass ratios of up to 60% without any chemical treatment on the filler has been validated.

Finally, this work studied the development of a 100% bio-sourced agrocomposite from all argan byproducts (shell, pulp, and argan cake) without any chemical treatment and BioPE as a matrix. It can be deduced that all the byproducts studied can be used as a bio-filler of the biocomposites due to their mechanical performance. Indeed, in the majority of cases, only the argan shell was studied as a filler and it was always chemically treated, thus the maximum mass ratio added was about 25% [36,43,61].

## 4. Conclusions

The adding of Moroccan argan byproducts as fillers in the polymer matrix represents promising new and eco-friendly products, with high performance and low cost for such industrial application as packaging. The present study deals with the thermal, structural, and mechanical properties of argan byproducts (shell, pulp, and cake) as a new bio-filler. The high-performance mechanical properties of the used bio-fillers (Young’s modulus and tensile strength) yielded an improvement in the mechanical properties of the final composite compared to those of the pure BioPE. Overall, the use of shell, pulp and argan cake particles shows potential for applications in the plastics industry due to their ability to yield improved mechanical properties in a cost-effective manner. Argan byproducts used as bio-filler appear to be a good alternative for obtaining environmentally friendly products.

## Figures and Tables

**Table 1:** Content of main chemical constituents of argan byproducts (pulp, cake, and shell).

Composition %*_w/w_*	Pulp	Roasted Cake	Unroasted Cake	Shell
Dry matter	5.02 ± 0.16	3.48 ± 0.11	3.42 ± 0.10	5.02 ± 0.13
Ash	0.29 ± 0.01	0.20 ± 0.01	0.21 ± 0.01	3.20 ± 0.01
Fat	5.46 ± 0.26	7.8 ± 0.22	7.01 ± 0.20	1.02 ± 0.16
Protein	5.02 ± 0.16	38.1 ± 0.1	36.7 ± 0.6	3.46 ± 0.12
Cellulose	19.35 ± 0.14	17.6 ± 0.1	17.1 ± 0.1	46.15 ± 0.12
* NDF/total fibers	29.26 ± 0.19	21.2 ± 0.1	21.1 ± 0.1	94.58 ± 0.15
** ADF Lignocellulose	22.52 ± 0.05	17.38 ± 0.03	17.36 ± 0.04	75.58 ± 0.10
Hemicellulose	6.75 ± 0.10	3.82 ± 0.11	3.85 ± 0.11	19.06 ± 0.10
Lignin	3.17 ± 0.12	2.78 ± 0.10	2.75 ± 0.12	29.17 ± 0.14

* NDF: Neutral-Digestible Fiber; ** ADF: Acid-Digestible Fiber.

**Table 2:** Distribution of argan byproduct granules.

Argan Byproducts	Granule Size (μm)				Span	Specific Surface Area (m²/kg)
	D_10_	D_50_	D_90_	D [4;3]		
Shell	18.0	62.2	144.0	73.0	2.0	217.9
Pulp	19.3	101.0	301.0	135.0	2.8	139.0
Roasted Argan cake	5.6	29.2	95.0	43.3	3.1	559.1
Unroasted Argan cake	4.7	26.2	131.0	53.0	4.8	646.7

**Table 3:** Thermal parameters determined using differential scanning calorimetry (DSC).

Polymer	ΔHm (J/g)	Tm (°C)	Tc (°C)	ΔHc (J/g)	Xc (%)
PE	111	133	112	114.03	39.6
PE 30% Shell	73	134	112	66.53	23.1
PE 30% Pulp	56	132	112	74.78	25.9
PE 30% Roasted Argan cake	32	132	112	79.48	27.6
PE 30% Unroasted Argan cake	31	133	112	87.77	30.5

**Table 4:** Summary of mechanical property results. Young’s modulus (E), maximum tensile strength (σ_max_), ultimate strain ε _max_ (%), and elongation at break (ε_b_).

Polymer	PE	PE 30% Shell	PE 30% Pulp	PE 30% Roasted Argan Cake	PE 30% Unroasted Argan Cake
E (Mpa)	206 ± 36	498 ± 39	349 ± 47	324 ± 31	330 ± 41
σ_max_ (Mpa)	10.15 ± 0.81	10.18 ± 0.57	9.58 ± 0.93	9.99 ± 0.67	9.99 ± 0.54
ε_max_ (%)	5.83 ± 0.1	4.28 ± 0.2	5.86 ± 0.1	4.94 ± 0.1	5.13 ± 0.3
ε_b_ (%)	26.75 ± 0.32	7.82 ± 0.12	11.34 ± 0.10	9.01 ± 0.10	19.67 ± 0.21

## Data Availability

Not applicable.
